# Translation and validation of the Chinese version of the Current Opioid Misuse Measure (COMM) for patients with chronic pain in Mainland China

**DOI:** 10.1186/s12955-015-0329-y

**Published:** 2015-09-15

**Authors:** Yang Zhao, Yuli Li, Xuekun Zhang, Fenglan Lou

**Affiliations:** School of Nursing, Shandong University, No.44, Wenhuaxi Road, Jinan, Shandong Province 250012 People’s Republic of China; Nursing Faculty, Tianjin Medical College, No. 14 Liulin Road, Tianjin, 300222 People’s Republic of China

## Abstract

**Background:**

Management of prescription opioids misuse and abuse problems among chronic pain patients has been increasingly important worldwide and little literature concerning prescription opioids can be found in mainland China so far.

**Methods:**

The Current Opioid Misuse Measure (COMM) was translated into Chinese following Brislin’s model of cross-culture translation and was completed by a convenience sample of 180 patients with chronic pain recruited from two major hospitals in Jinan, Shandong province. Data were analyzed using internal consistency, test-retest reliability, exploratory factor analysis and confirmatory factor analysis.

**Results:**

The internal consistency coefficient for the total score of the COMM was 0.85 and item-total correlations of all items were above 0.20. Besides, the test-retest reliability was satisfactory with an ICC of 0.91 (95 % CI = 0.65-0.98). Four principal components were extracted, accounting for 65.30 % of the variance, and the factor loadings of all 17 items were above 0.40.

**Conclusions:**

The Chinese version of COMM showed satisfactory reliability and validity, and could be used as a screening tool to evaluate and monitor current aberrant drug-related behavior among Chinese patients with chronic pain.

## Background

Opioids, as a potentially effective treatment for chronic pain, have increasingly been prescribed for pain relief among patients with chronic pain in recent years. Despite the benefits of opioid therapy, opioid-related problems are also increasing. In Australia, oxycodone prescriptions increased, particularly among older patients [1]. In the United States, prevalence of chronic opioid use increased from 3 % in 2003 to 4.5 % in 2007, and multiple studies in the literature have reported an association between opioid prescribing and overall health status, with increased disability, medical costs, subsequent surgery, and continued or late opioid use [2]. Likewise, in Canada, according to available data, the prescription opioid consumption levels were increased and high. Correspondingly, its nonmedical prescription opioid use and prescription opioid related harms were high, and may now constitute the third highest level of substance use burden of disease [3]. In addition, Dhalla found that the prescribing of opioid analgesics in Ontario increased by 29 %, from 458 to 591 prescriptions per 1000 individuals annually between 1991 and 2007, and the annual number of opioid-related deaths increased by 41 %, from 19.4 to 27.2 per million annually between 1999 and 2004 [4]. Therefore, identifying which patients under opioid treatment may develop opioid-related aberrant behaviors is important. Meanwhile, management of pain medication misuse and abuse problems poses significant challenges. However, in Mainland China, little is known about the prescription opioids among chronic pain patients. From our own experience as well as consulting clinical experts, we get to know that opioids such as codeine, oxycodone, tramadol are often therapeutically prescribed for pain relief in China. However, physicians usually lay emphasis on pain treatment, in view of the sever impairment both physically and psychologically brought by chronic pain, like activity limitation in daily life, depression, anxiety and anger, which leads them to ignore the potential aberrant drug-related behaviors occurring during the process of pain treatment. Therefore, such phenomenon arouse our attention and inspire our interests on conducting this research, with the aim of introducing an opioid screening tool and investigating the current status of aberrant drug-related behavior in Chinese patients with chronic pain.

“Misuse” is defined as use of the substance not according to medical indications or prescribed dosing. In addition, misuse is specifically restricted to the prescription or over-the-counter medications, and it occurs only when a drug is taken for medical purpose [5]. At present, there are several ways available to help detect drug misuse, such as urine drug testing (UDT), psychosocial screening interviews and screening tools (self-report or clinician rated) [6]. The screening tools were convenient, time-saving and cost-effective, and therefore they were important and indispensable opioid monitoring methods. Among these tools, the Screener and Opioid Assessment for Patients with Pain (SOAPP) was intended to be used at an initial visit or when considering treatment with an opioid, the Prescription Drug Use Questionnaire (PDUQ) and the Pain Assessment and Documentation Tool (PADT) were observational screening methods, the Opioid Risk Tool (ORT) was susceptible to deception and fits validity is not high. Finally, we chose the Current Opioid Misuse Measure (COMM) as the tool in our research [7–11].

The COMM was developed to assess current misuse behaviors for patients with chronic pain who has been on opioid therapy for an extended period of time. Initially, pain management and addiction specialists developed a 40-item alpha COMM using concept mapping, which was then tested in 227 patients taking opioid for chronic, noncancer pain and retested one week later with 55 of them. Meanwhile, all participants finished the Prescription Drug Use Questionnaire (PDUQ) as well as urine drug testing for toxicity screening. Finally, 17 items demonstrated a capacity to measure aberrant behavior with good validity and reliability (Cronbach's α = 0.86; ICC = 0.86, 95 % CI: 0.77-0.92). The cutoff score of the COMM was 9. Three months later, 86 patients were re-assessed and showed adequate ability of the COMM to track changes over time [12]. Furthermore, through cross validation, Butler reported an excellent internal consistency and validity of the COMM with a coefficient alpha being 0.83 and an AUC of 0.79 (Standard error = 0.031; 95 % CI: 0.73-0.85) [13]. Meltzer found that the sensitivity and specificity of the COMM were both 0.77 to identify patients with prescription drug use disorder (PDD) when using the DSM-IV criteria for PDD as the “gold standard” and the cutoff score was 13 [14]. It is a useful tool for clinicians to periodically monitor misuse of opioid medication and to develop treatment strategies in order to minimize continued misuse [12]. Besides, current practice guidelines recommend using the COMM to assess patients receiving prescription opioid therapy [15].

The aim of this study was to: (1) translate the COMM into Chinese; (2) investigate the reliability and validity of the COMM among Chinese-speaking patients with chronic pain; (3) determine whether the COMM can be used in a Chinese population.

## Methods

### Participants and settings

Participants were recruited from pain clinics in two major hospitals between July and October 2014 in Jinan city, Shandong Province, China. The inclusion criteria were: (1) age 18 years or older; (2) chronic pain for 3 months or longer; (3) prescription opioid medications for 1 month or longer; and (4) ability to read and speak Chinese with no significant or diagnosed cognitive problems. Ethical approval was obtained from the Research Ethics Committees in Shandong University. In addition, informed consent was obtained from each participant and they had the right to choose whether they would complete the survey independently or have the researcher read the questions and record their responses.

### The instrument

The COMM is a validated 17-item instrument, developed by Butler et al. to assess past-month aberrant medication-related behaviors for pain patients taking opioids [12]. A 5-point Likert scale ranging from 0 (never) to 5 (very often) is used for each item and the COMM total score is the sum of the 17 items. As a self-report screening instrument, the COMM provides an estimate of the patients “current” status and its items capture a 30-day period. It appears to be a reliable and valid screening tool to monitor misuse behaviors among chronic pain patients [13, 14]. Furthermore, Finkelman et al. reported that curtailment and stochastic curtailment of the COMM reduced its respondent burden without compromising sensitivity and specificity [16].

Demographic information was obtained from the questionnaire we designed to collect demographic and pain-related information of the participants, including age, sex, education, marital status, pain duration, months of opioid treatment and pain location.

### Translation process

Content licensing agreement was obtained from the original authors. The COMM was translated into Chinese, following Brislin’s model [17, 18]. The translation of the COMM from original English to Chinese (COMM-A and COMM-B) was first performed by two independent and professional translators (A and B) in the research team. The two translation versions (COMM-A and COMM-B) were merged into one single forward translation version (COMM-A/B) by a third professional and native speaker (C). This version was translated back into Chinese by a forth bilingual researcher (D) who had never seen the scale previously. Discrepancies between the original COMM and the back-translated version were reviewed for equivalence of meaning. Lastly, the Chinese translation version of COMM was modified and polished.

The translated COMM was pilot-tested on a convenience sample of twenty pain patients. After the test, new problems concerning clarity, comprehension and interpretability were discussed. For example, the “road rage” was seldom used in China, many patients had difficulty understanding this phrase, and then we discussed how to express it more clearly and more comprehensible. Finally, the Chinese version COMM was obtained when no substantial disagreements could be found, as is shown in Table 1.

**Table 1:** Items of the Chinese version of COMM

Item Number	English Items	Items Translated Into Chinese
1	How often have you had trouble with thinking clearly or had memory problems?	思维不清或出现记忆方面的问题
2	How often do people complain that you are not completing necessary tasks? (i.e., doing things that need to be done, such as going to class, work, or appointments)	周围的人抱怨您不能完成一些重要的事情(例如上课、上班、约会等)
3	How often have you had to go to someone other than your prescribing physician to get sufficient pain relief from your medications? (i.e., Another doctor, the Emergency Room)	为得到足量的止疼药而找其他的医生或去急诊室
4	How often have you taken your medications differently from how they are prescribed?	不按照医嘱服用止疼药
5	How often have you seriously thought about hurting yourself?	有自残的想法
6	How much of your time was spent thinking about opioid medications (having enough, taking them, dosing schedule, etc.)?	思考与止疼药有关的问题(例如是否足够、怎样服用、剂量如何等)
7	How often have you been in an argument?	与别人争吵
8	How often have you had trouble controlling your anger (e.g., road rage, screaming, etc.)?	控制不住自己的脾气(例如交通阻塞时产生愤怒情绪、大声争吵等)
9	How often have you needed to take pain medications belonging to someone else?	服用其他人的止痛药
10	How often have you been worried about how you’re handling your medications?	担忧自己服用止疼药的相关事宜(例如服药方式、剂量等)
11	How often have others been worried about how you’re handling your medications?	令他人担忧自己服用止疼药的相关事宜(例如服药方式、剂量等)
12	How often have you had to make an emergency phone call or show up at the clinic without an appointment?	拨打急救电话或者不按照预约的时间到医院就诊
13	How often have you gotten angry with people?	迁怒于别人而发脾气
14	How often have you had to take more of your medication than prescribed?	服用止疼药的剂量超过医嘱的要求
15	How often have you borrowed pain medication from someone else?	借用别人的止疼药
16	How often have you used your pain medicine for symptoms other than for pain (e.g., to help you sleep, improve your mood, or relieve stress)?	出于除疼痛之外的其他原因而服用止疼药(例如催眠、改善情绪或减压)
17	How often have you had to visit the Emergency Room?	到医院急诊室就医

A panel of experts was invited to evaluate the content equivalence of the COMM using content validity index (CVI), which is a 4-point rating scale (1 = not relevant; 2 = somewhat relevant; 3 = quite relevant; 4 = highly relevant). The CVI is the percentage of agreement of all items rated by the experts as either three or four and an ICC of 0.8 or greater is generally considered to be an indication of good content validity [19].

### Statistical analyses

Statistical analyses were conducted using SPSS version 19.0 and Amos version 17.0. Statistical significance was set at *P* < 0.05. For categorical variables, data were presented in forms of frequencies and percentages, and continuous variables were reported as means ± standard deviation (SD).

The reliability of the COMM was assessed by internal consistency and test-retest reliability. Specifically, the internal consistency reliability was determined by using Cronbach’s alpha coefficients, corrected item-total correlation and inter-item correlation matrix analysis. A Cronbach’s alpha of ≥0.70 and item-total correlation of >0.2 were deemed statistically acceptable [20]. The test-retest reliability was evaluated by calculating the intra-class correlation coefficients (ICC) using data from 10 patients who filled out the COMM within the 2-week interval. An ICC of 0.8 or higher was to be considered acceptable [21].

Construct validity for the COMM was evaluated by principal components analysis with Promax rotation. We first conducted the Kaiser-Meyer-Olkin (KMO) measure and Bartlett’s test to determine if there were a statistically significant correlation among items to perform this analysis [22]. A value of 0.40 or greater for the factor loadings was regarded as acceptable [23]. Furthermore, confirmatory factor analysis (CFA) was conducted to compare the hypothesized factor structure with the research data. Specifically, various well-established model fit indices including Chi square test, Chi square/df ratio, root mean square error of approximation (RMSEA), comparative fit index (CFI), incremental fit index (IFI), Tucker-Lewis index (TLI), root of the mean square residual (RMR), goodness-of-fit index (GFI), and adjusted GFI (AGFI) were used to assess the model fit.

## Results

### Sample characteristics

The 180 chronic pain patients (155 non-cancer and 25 cancer pain patients) had an average age of 55.74 years (SD 16.36), with a range of 18–88 years, and 52.22 % were male. Overall, seventy four patients (41.11 %) had received more than nine years’ education and 155 participants (86.11 %) were married. The average duration of pain was 27.08 months (SD = 52.42), with a range of 3–360 months. The most common primary pain locations were lumbar or sacral pain (38.33 %) and leg pain (35.00 %), as is shown in Table 2.

**Table 2:** Demographic characteristics of the participants (*n* = 180)

Characteristics	*n* (%)	Mean (SD)	Median
Age (years)		55.74 (16.36)	59.00
Gender			
Male	94 (52.22)		
Female	86 (47.78)		
Education			
≤1 year	21 (11.67)		
≤5 years	27 (15.00)		
≤9 years	58 (32.22)		
>9 years	74 (41.11)		
Marital status			
Married	155 (86.11)		
Single	15 (8.33)		
Separated/divorced	4 (2.22)		
Widowed	5 (2.78)		
Pain duration (months)		27.08 (52.42)	5.00
Months of opioid treatment		8.83 (30.35)	2.00
Pain locations			
Head, face	22 (12.22)		
Cervical	24 (13.33)		
Shoulder, arm	21 (11.67)		
Thorax	26 (14.44)		
Abdomen	15 (8.33)		
Back	24 (13.33)		
Lumbar, sacral	69 (38.33)		
Pelvic	29 (16.11)		
Leg	63 (35.00)		

### Reliability

The Cronbach’s alpha and the corrected item-total correlations of all 17 item are shown in Table 3. The internal consistency coefficient (Cronbach’s α) for the total score of the COMM was 0.85. Item-total correlations ranged from 0.24 (item 9) to 0.62 (item 14) and all items met the recommended minimum of 0.20. In addition, as is shown in Table 4, some items showed poor or negative inter-item correlation, suggesting that COMM may not be a unidimensional measurement.

**Table 3:** Mean scores, corrected item-total correlations of the Chinese version of COMM

Item	Mean	SD	Corrected item-total correlation	Cronbach’s alpha if item deleted
1	0.70	0.94	0.38	0.84
2	0.62	0.92	0.52	0.83
3	0.40	0.77	0.30	0.84
4	0.54	0.75	0.52	0.83
5	0.34	0.71	0.53	0.83
6	1.15	1.07	0.38	0.84
7	0.92	0.86	0.54	0.83
8	1.21	1.04	0.54	0.83
9	0.17	0.52	0.24	0.84
10	0.97	1.03	0.42	0.84
11	0.77	0.95	0.53	0.83
12	0.23	0.61	0.42	0.84
13	1.15	1.11	0.57	0.83
14	0.57	0.89	0.62	0.83
15	0.12	0.39	0.29	0.84
16	0.35	0.71	0.45	0.84
17	1.83	1.45	0.52	0.84

**Table 4:** Correlation matrix of COMM items

Item	1	2	3	4	5	6	7	8	9	10	11	12	13	14	15	16	17
1	1																
2	0.65**	1															
3	0.10	0.19*	1														
4	0.13	0.25**	0.27**	1													
5	0.24**	0.41**	0.19**	0.27**	1												
6	0.12	0.17*	0.22**	0.23**	0.24**	1											
7	0.24**	0.26**	0.05	0.22**	0.34**	0.10	1										
8	0.22**	0.27**	0.04	0.30**	0.39**	0.09	0.76**	1									
9	−0.07	0.03	0.44**	0.27**	0.18*	0.11	0.07	0.06	1								
10	0.08	0.20**	0.11	0.14	0.29**	0.67**	0.17*	012	0.11	1							
11	0.20**	0.35**	0.24**	0.24**	0.27**	0.50**	0.25**	0.21**	0.17*	0.70**	1						
12	0.33**	0.26**	0.20*	0.24**	0.20**	0.10	0.28**	0.29**	0.03	0.07	0.16*	1					
13	0.26**	0.26**	−0.01	0.33**	0.40**	0.13	0.73**	0.84**	0.05	0.15	0.24**	0.29**	1				
14	0.14	0.28**	0.36**	0.69**	0.33**	0.22**	0.29**	0.34**	0.39**	0.22**	0.30**	0.33**	0.39**	1			
15	−0.01	0.05	0.34**	0.38**	0.16*	0.09	0.07	0.07	0.60**	0.12	0.20*	0.07	0.04	0.51**	1		
16	0.260**	0.40**	0.28**	0.36**	0.33**	0.25**	0.13	0.03	0.23**	0.24**	0.250**	0.28**	0.11	0.48**	0.24**	1	
17	0.32**	0.38**	0.11	0.34**	0.27**	0.15*	0.41**	0.41**	0.01	0.20**	0.27**	0.38**	0.43**	0.42**	0.05	0.25**	1

*P < 0.05. **P <0.01

Moreover, of the 180 participants, 10 were randomly selected to evaluate the teat-retest reliability in two weeks’ interval. The mean scores of the first and second measurements were 13.37 ± 8.15 and 17.80 ± 4.92, respectively. The test-retest reliability was satisfactory with an ICC of 0.91 (95 % CI = 0.65-0.98).

### Validity

For the COMM, all of the 17 items showed a CVI above 0.8, which indicates that the construct validity of the COMM was acceptable.

The construct validity of the COMM was determined by principal components factor analysis with Promax rotation. The result of the KMO measure was 0.78, and the approximate chi-square for Bartlett’s test was 1390.099 (df = 136, *P* < 0.001). As shown in Table 5, the factor loadings of all 17 items ranged from 0.42 (item 5) to 0.96 (item 8). Four principal components were extracted with eigenvalue of 4.97, 2.51, 1.98 and 1.63, respectively. Variables 5, 7, 8 and 13 load on factor 1; 3, 4, 9, 14 and 15 load on factor 2; 1, 2, 12, 16 and 17 load on factor 3; and 6, 10 and 11 load on factor 4. The percent variances for the four factors were 29.25, 14.79, 11.65 and 9.61 %, respectively.

**Table 5:** Factor analysis results for the Chinese version of COMM

Item	Factor 1	Factor 2	Factor 3	Factor 4
8	**0.96**	−0.04	−0.06	−0.03
13	**0.94**	−0.02	−0.05	0.05
7	**0.88**	−0.06	0.01	0.01
5	**0.42**	0.16	0.18	0.15
15	−0.50	**0.86**	−0.22	−0.05
9	−0.51	**0.84**	−0.24	0.02
14	0.23	**0.73**	0.10	−0.03
4	0.20	**0.63**	0.12	−0.01
3	−0.26	**0.63**	0.19	0.04
1	−0.05	−0.26	**0.91**	−0.04
2	−0.04	−0.10	**0.88**	0.07
12	0.13	0.12	**0.55**	−0.15
16	−0.22	0.38	**0.53**	0.08
17	0.22	0.18	**0.50**	−0.06
10	0.05	−0.08	−0.09	**0.97**
6	−0.06	0.01	−0.04	**0.87**
11	0.05	0.06	0.08	**0.80**
Eigenvalue	4.97	2.51	1.98	1.63
Percent variance (%)	29.25	14.79	11.65	9.61

A value of 0.40 or greater for the factor loadings was regarded as acceptable

CFA was performed to test the four-factor model, and the results showed that it did not exhibit acceptable fit indexes. Then modification was made to improve the fit. Finally, an acceptable model fit was set, with a four-factor structure. The modified four factors were: Factor 1 (item 5, 7, 8 and 13), Factor 2 (item 4, 9, 14 and 15), Factor 3 (item 1, 2, 3, 12, 16 and 17), and Factor 4 (item 6, 10 and 11). According to the findings from the CFA, which are shown in Table 6, the model fitting was acceptable [24].

**Table 6:** Results of the confirmatory factor analysis

*Χ* ^2^	*Χ* ^2^/df	RMSEA^a^	CFI^a^	IFI^a^	TLI^a^	RMR^a^	GFI^a^	AGFI^a^
250.08	2.55	0.05	0.88	0.90	0.91	0.06	0.93	0.90

^a^ *RMSEA*, root mean square error of approximation; *CFI*, comparative fit index; *IFI*, incremental fit index; *TLI*, Tucker-Lewis index; *RMR*, root of the mean square residual; *GFI*, goodness-of-fit index; *AGFI*, adjusted GFI

## Discussion

To our knowledge, this study is the first to apply screening tool of opioid medication to assess aberrant drug-related behaviors in chronic pain patients in Mainland China. Besides, it also provides the first report on the translation and validation of the COMM into Chinese language. The results in this study show good qualities of the COMM in terms of content, interpretability and acceptability. We are able to show feasibility for the use of the Chinese version of COMM to measure misuse behaviors of prescription opioid among patients with chronic pain.

The Cronbach’s coefficient alpha of the Chinese version of COMM exceeded 0.70, the general requirements of a questionnaire internal consistency. This value was approximately equal to that of the original English version [12]. The item-total correlations reached the general requirement of 0.20. In addition, ‘Cronbach’s alpha if item deleted’ showed that when an item was deleted from the COMM, the total questionnaire Cronbach’s coefficient alpha would not significantly changed, which suggests that its items had good internal consistency reliability. Besides, at an individual questionnaire level, some items do not correlate well with each other. It might be due to the fact that according to the original version, all 17 items came from five clusters identified through the concept mapping process. With regard to test-retest reliability, the ICC value for the COMM reached more than 0.80, indicating good agreement. And the ICC value in our study was higher than the result reported by Butler et al. These findings demonstrate that the Chinese version of COMM is a reliable brief measure for assessing aberrant drug-related behavior.

The CVI was excellent, showing that all seventeen items evaluate the same construct as an overall instrument. In the original article of the COMM development, all the 17 items came from five clusters generated during the brainstorming stage, they are: (1) Signs & Symptoms of Drug Misuse (item 1); (2) Emotional Problems/Psychiatric Issues (item 2, 5, 7, 8 and 13); (3) Appointment Patterns (item 3, 12 and 17); (4) Evidence of Lying and Drug Use (item 4, 6, 9, 10 and 11); (5) Medication Misuse & Noncompliance (item 14, 15 and 16) [12]. According to the results of factor analysis, all the 17 items of the COMM were grouped into the four factors, with factor loadings reaching the criteria of 0.40. It showed that the construct of the Chinese version of COMM did not keep in with what originally intended. Specifically,the first factor was labeled emotional or psychiatric problems (item 5, 7, 8 and 13), the second was medication misuse or noncompliance (item 4, 9, 14 and 15), the third was memory and doctor visit (item 1, 2, 3, 12, 16 and 17), and the fourth was apprehension of drug use (item 6, 10 and 11). These factors indicated the four possible underlying dimensions of the COMM and these factors explained 65.30 % of the variance. According to the two-index presentation format suggested by Hu and Bentler, when RMSEA was 0.06 or lower and SRMR was 0.09 or lower, the model fit was acceptable. The fitting indexes from CFA fully proved that the model fitting was acceptable [24].

Although, aberrant drug-related behaviors occurring during the process of opioid treatment have increasingly been social and ethical problems for us to deal with, it is important to note that the COMM is not meant to be used punitively, or to deny access to pain relief. It is just one measurement to assess potential opioid misuse for patients on opioid therapy, with the aim of achieving the best opioid treatment effect and then improving the quality of life.

There are several limitations in the present study. The researchers in our research team, the funds we got, and the time of conducting this study were all limited, so the sample was recruited only from two major hospitals in Jinan city, Shandong province, in which the pain clinics were ones with relatively larger scale. The two hospitals we chose were both tertiary A hospitals and located in urban area. In addition, all the patients in the present study were not selected randomly, and 65.14 % of them came from cities, the most common primary pain locations were lumbar or sacral and leg pain, accounting for 73.33 %, and codeine, oxycodone, tramadol were more used prescription opioids. Therefore, the findings may not be extrapolated to the general chronic pain patients. Further studies from diverse populations across geographic locations are needed for generalization of the findings to the whole chronic pain patients taking prescription opioid. Moreover, criterion validity of the COMM was not analyzed, since no Chinese version tools, which could be used as proper “criterion” to assess participants' drug misuse behaviors, could be found. However, other methods including UDT and psychosocial screening interviews, could be used as references in combination with the Chinese version of COMM to evaluate its predictive and diagnostic capabilities in further studies. Besides, because limitations of time, financial and human resources, the sample size for a test-retest reliability testing was quite small, and the sample size for the CFA is not big as well. Further research should recruit a much larger sample to verify our research findings. Last but not least, other screening tools for aberrant drug-related behaviors, either self-report or physician rated, such as the pain medication questionnaire (PMQ), screener and opioid assessment for patients with pain (SOAPP), opioid risk tool (ORT), prescription drug use questionnaire (PDUQ) and pain assessment and documentation tool (PADT) [7–11, 25], should also be introduced, explored and compared with the COMM in their properties and characteristics in order to provide a more effective and efficient way for management of pain medication.

## Conclusions

In conclusion, this is the first attempt to translate and validate the COMM in a non-English-speaking country. As reported in this study, the Chinese version of COMM demonstrated relatively high level of reliability and validity among Chinese patients with chronic pain. This self-report measure could evaluate and monitor current aberrant drug-related behavior as a screening tool among Chinese patients with chronic pain-prescribed opioids.

## Abbreviations

COMM: Current Opioid Misuse Measure
ICC: Intra-class correlation coefficient
AUC: Receiver operating characteristic curve
UDT: Urine drug testing
PDD: Prescription drug use disorder
CVI: Content validity index
CI: Confidence intervals
KMO: Kaiser-Meyer-Olkin
SD: Standard deviation
CFA: Confirmatory factor analysis
RMSEA: Root mean square error of approximation
CFI: Comparative fit index
IFI: Incremental fit index
TLI: Tucker-Lewis index
RMR: Root of the mean square residual
GFI: Goodness-of-fit index
AGFI: Adjusted GFI
